# Do we need MRI in all biopsy naïve patients? A multicenter cohort analysis

**DOI:** 10.1007/s00345-024-04780-1

**Published:** 2024-02-07

**Authors:** Philipp Krausewitz, Angelika Borkowetz, Gernot Ortner, Kira Kornienko, Mike Wenzel, Niklas Westhoff, GESRU Academics Prostate Cancer Group

**Affiliations:** 1 Department of Urology and Pediatric Urology, University Medical Center Bonn (UKB), University Hospital Bonn, Bonn, Germany; 2 Department of Urology, University Hospital Carl Gustav Carus, Technische Universität Dresden, Dresden, Germany; 3 Department of Urology, LKH Hall, Hall in Tirol, Austria; 4 Department of Urology, Charité University Medicine Berlin, Berlin, Germany; 5 Department of Urology, University Hospital Frankfurt, Goethe University Frankfurt, Frankfurt, Germany; 6 Department of Urology and Urological Surgery, University Medical Center Mannheim, Medical Faculty Mannheim, University of Heidelberg, Mannheim, Germany

**Keywords:** Clinically significant prostate cancer, Biopsy method, MRI, Systematic biopsy

## Abstract

**Purpose:**

The combined approach (CB) of magnetic resonance imaging (MRI)-guided biopsy (TB) and systematic biopsy (SB) is strongly recommended based on numerous studies in biopsy naïve men with suspicion of clinically significant prostate cancer (csPCA). However, the unbalanced accessibility of MRI, challenges related to reimbursement and the scarcity of specialized medical practitioners continue to impede a widespread implementation.

Therefore, our objective was to determine a subset of men that could undergo SB without an increased risk of underdiagnosis at reduced expenses.

**Methods:**

A multicenter analysis of 2714 men with confirmed PCA and suspicious MRI who underwent CB were enrolled. Cancer detection rates were compared between the different biopsy routes SB, TB and CB using McNemar paired test. Additionally, Gleason grade up- and down-grading was determined.

**Results:**

CB detected more csPCA than TB and SB (*p* < 0.001), irrespective of MRI findings or biopsy route (transperineal vs. transrectal). Thereby, single biopsy approaches misgraded > 50% of csPCA. TB showed higher diagnostic efficiency, defined as csPCA detection per biopsy core than CB and SB (*p* < 0.001). For patients with abnormal DRE and PSA levels > 12.5 ng/ml, PSAD > 0.35 ng/ml/cm^3^, or > 75 years, SB and CB showed similar csPCA detection rates.

**Conclusion:**

Conducting CB provides the highest level of diagnostic certainty and minimizes the risk of underdiagnosis in almost all biopsy-naive men. However, in patients with suspicious DRE and high PSA levels, PSAD, or advanced age solely using SB leads to similar csPCA detection rates. Thus, a reduced biopsy protocol may be considered for these men in case resources are limited.

## Introduction

The accurate differentiation between clinically significant prostate cancer (csPCA) and clinically non-significant prostate cancer (nsPCA) is crucial in determining the appropriate treatment approach for patients. However, the heterogeneity of prostate tumors makes risk stratification challenging. Due to the use of the prostate imaging reporting and data system (PI-RADS) in multiparametric magnetic resonance imaging (MRI) PCA is detected significantly better during initial and re-biopsy with a significant reduction in overdiagnosis of nsPCA and a lower number of biopsies compared to the systematic biopsy (SB) method [1–3]. Despite its usefulness, the MRI-targeted biopsy (TB) is not without limitations. Notably, it may fall short in detecting a significant proportion of csPCA [4]. The reasons for this are manifold. First, technical limitations in diffusion-weighted sequences and the significant variability in interpretation by radiologists can lead to inaccurate image acquisition, registration and analysis [5, 6]. Second, the performance of TB is more complex compared to SB, as it involves multiple steps by at least two proficiencies including interdisciplinary communication with structured reporting, marking suspicious regions, precise ultrasound navigation and targeted biopsy sampling [7, 8]. In addition, learning curves of health care providers have to be considered [9, 10]. As a result, the negative predictive value of the prostate MRI is heterogenic and does not justify avoiding biopsy if MRI results are negative in general [11]. Moreover, the positive predictive value of the MRI for csPCA is variable and low (40%). Therefore, SB cannot be omitted in biopsy naïve men and additional individual risk assessment is still needed and recommended [12, 13]. Obstacles on a macrolevel are the access and costs for the infrastructure required including the necessary equipment and qualified medical professionals leading to disparities in healthcare access and outcomes. In keeping with these findings, there are still significant barriers to make the advanced technique of TB accessible to a wide population despite clear advantages regarding cancer detection rates (CDR) and rising evidence for cost-effectiveness of the procedure [14, 15].

The study aimed to investigate two objectives: firstly, to assess the necessity of incorporating MRI prior to initial biopsy in a multicenter cohort, and secondly, to investigate clinical measures that can predict the safety of a reduced biopsy approach using only SB in a subset of patients.

## Patients and methods

### Patients

Within this multicenter project, biopsy-naïve men who underwent CB with concordant PCA diagnosis were gathered from six high-volume centers by the German Society of Residents in Urology Academics (*n* = 2874). Indications for MRI were suspicious Prostate-specific Antigen (PSA), abnormal digital rectal examination (DRE) and/or abnormal findings on transrectal ultrasound (TRUS). Men with PSA > 20 ng/ml (*n* = 133) and resulting very high probability for advanced and/or suspected metastatic disease [16, 17] were excluded from primary analysis in order to prevent pre-analytical dominance bias and analyzed supplementary. Hence, *n* = 2714 men with confirmed PCA and PI-RADS 3–5 graded lesions were analyzed.

*Biopsy Process*: Before the biopsy, all patients underwent MRI, which was interpreted according to PI-RADSv2 by board-certified radiologists at each center. A software-based transrectal (TR, *n* = 1951) or transperineal (TP, *n* = 763) CB including a standardized 12-core SB was performed by board-certified urologists or supervised residents, following consensus recommendations. All centers utilized software-assisted fusion techniques. Biopsy cores were individually documented, collected, and histopathologically evaluated according to guidelines [13].

### Analysis & statistics

csPCA was defined as ≥ Gleason 3 + 4 (International Society of Urological Pathology (ISUP) Grade Group ≥ 2). CDR of SB, TB, and CB were compared, stratified by PI-RADS score, age, prostate volume, DRE, and PSA density (PSAD). The subset of patients with divergent biopsy results in terms of tumor detection and grading were identified, Supplementary Fig. S1. All data were coded and analyzed with "IBM SPSS Statistics," v27 Armonk, NY: IBM Corp 2020. Descriptive statistics included frequencies and proportions for categorical variables. Means and standard deviation were reported for continuously coded variables. Differences were detected using the T test for independent samples, chi-square tests or McNemar paired test. Binary logistic regression was used in both univariate and multivariate analyses to determine significant csPCa predictors. Diagnostic accuracy was described by receiver operating characteristic curve (ROC) analysis and the area under the curve (AUC) was computed. *P* values of ≤ 0.05 were considered statistically significant.

## Results:

### Combined vs. single procedure

csPCA was detected in 72.7% of all cases including 21.0% of high-risk PCA, defined as Gleason ≥ 8. Overall CDR by CB for PCA, csPCA and high-risk PCA was significantly higher than those of TB and SB (all *p* < 0.001). PI-RADS 3–5 distribution was 13%, 54% and 33.0% with corresponding tumor detection rates of 55%, 70%, and 84%. The superiority of CB over single procedures regarding csPCA detection was also shown in a PI-RADS-dependent comparison in the groups 3–5 (all *p* < 0.001, Supplementary Fig. S2). Detailed patient’s characteristics are shown in Table 1. Analyses regarding the value of PCA surrogate markers in the cohort are presented in the supplements.

**Table 1:** Patients characteristics

Variable	All men (*n* = 2714)
Age (years)	67.7 ± 8.3
PSA (ng/ml)	7.4 ± 3.7
PSAD (ng/ml/cm^3^)	0.19 ± 0.13
Prostate volume (cm^3^)	47.1 ± 24.2
Abnormal DRE (%)	31.0
PI-RADS 3 (%)	13
PI-RADS 4 (%)	54
PI-RADS 5 (%)	33
Target lesions per patient	1.4 ± 0.7
Size of index lesion (mm)	13.9 ± 7.0
TB cores per patient	3.8 ± 2.2
TB cores per index lesion	2.8 ± 1.2
TP biopsy route	763 (28.1)
TR biopsy route	1951 (71.9)
CDR SB:	
PCA (%)	91.3
csPCA (%)	60.6
hrPCA (%)	17.4
nsPCA (%)	30.7
CDR TB:	
PCA (%)	83.1
csPCA (%)	60.9
hrPCA (%)	15.2
nsPCA (%)	22.2
CDR CB:	
PCA (%)	100
csPCA (%)	72.7
hrPCA (%)	21.0
nsPCA (%)	27.2

Table shows means and standard deviation or valid percentages of the collected patient data

*PSA* prostate specific antigen, *PSAD* prostate specific antigen density, DRE digital rectal examination, *PI-RADS* The Prostate Imaging-Reporting and Data System Version 2 (PI-RADS™ v2.1), *TP* transperineal, *TR* transrectal, *PCA* prostate cancer, *csPCA* clinically significant prostate cancer defined as Gleason ≥ 3 + 4, *hrPCA* high-risk PCA defined as Gleason ≥ 8, nsPCA non-clinically significant caner defined as Gleason = 6

### TB vs. SB

In a head to head comparison, CDR was significantly higher for SB (91.3%) than TB (83.1%), *p* < 0.001. However, csPCA detection was similar (*p* = 0.754). TB detected 8.6% less nsPCA than SB (22.2% vs. 30.7%, *p* = 0.001). In terms of efficiency, defined as CDR per biopsy core taken, TB was significantly more efficient for PCA and csPCA detection than SB (both *p* < 0.001). In a PIRADS-dependent comparison, however, csPCA were more frequently detected by SB in PI-RADS-3-rated patients, comparably frequently detected by SB and TB in PI-RADS-4-rated patients, and more frequently detected in TB in the PI-RADS 5 group (*p* = 0.001, *p* = 0.680, *p* = 0.002, respectively), Supplementary Fig. S2; Table S1.

### Supplementary analysis of men with PSA > 20 ng/ml

In this cohort, patients exhibited a mean PSA level of 58 ng/ml with a maximum of 912 ng/ml, suggestive of an increased probability of tumor presence, including metastasis. Notably, 95% of patients presented with csPCA, with SB yielding accurate diagnoses in 87% of these cases. Nevertheless, even within this subset, CB outperformed SB significantly (*p* < 0.001), while SB and TB displayed similar performance (*p* = 0.442). Regarding patients with abnormal palpation, CB's diagnostic capacity was comparable to that of SB (CDR csPCA SB 94.0% vs. CB 98.8%; *p* = 0.125). Further information on this subgroup is available in Supplementary Table S4.

### Concordance of inter-method tumor grading in CB

TB and SB simultaneously detected PCA in 2018/2714 (74.4%) patients, but tumor grading by TB and SB matched only in 1325/2714 (48.8%) men. Discrepant Gleason grading of both biopsy modalities were present in 51.2% of csPCA and in 58.0% of high-risk PCA cases. PCA, csPCA, high-risk PCA and nsPCA were solely diagnosed by SB in 16.9%, 9.4%, 5.1% and 36.9% of men. SB upgraded nsPCA to csPCA in 135/600 (22.5%) or high-risk PCA in 16/600 (2.7%) men. The incremental value of TB for PCA, csPCA and high-risk PCA detection was 8.7% (236/2714), 6.7% (133/1972), and 4.2% (24/571), respectively. TB diagnosed 103/742 (13.9%) additional men with nsPCA and upgraded nsPCA to csPCA in 197/833 (23.6%), or high-risk PCA in 18/833 (2.2%) cases. Interestingly, in cases where SB or TB detected nsPCA (739/2714), the complementary biopsy modality detected nsPCA in the majority of cases too (67.5% and 77.5%, respectively). Moreover, in cases where the ISUP grade was upgraded from 1 by the additional SB or TB, the majority of cases were found to be of a lower severity with a change in ISUP grade of 2 in 64.2% and 72.6%, respectively. In the multivariate analysis of nsPCA detected by SB, no variable could be significantly associated. On the other hand, in ISUP 1 cases detected by TB, reduced prostate volume and increased PI-RADS score were found to be predictive for overall cancer detection and detecting ISUP 1 PCA (*p* = 0.016, *p* = 0.03, respectively).

### TP vs. TR route

A comparison of biopsy methods showed that transperineal biopsies resulted in a higher detection rate of csPCA compared to transrectal biopsies (TP 78.8% (601/763) compared to 70.4% (1374/1951) for the TR route (*p* = 0.001; OR = 1.56; 95% CI, 1.28–1.90). In both biopsy routes CB was superior to single procedures (all *p* < 0.001). Specifically, when using TP, both TB and SB yielded detection rates of 65.1% (497/763) and 62.5% (477/763), respectively, whereas with TR, TB and SB resulted in detection rates of 59.3% (1157/1951) and 59.9% (1168/1951), respectively, Supplementary Fig. S4.

### SB vs. CB

**Fig. 1:**
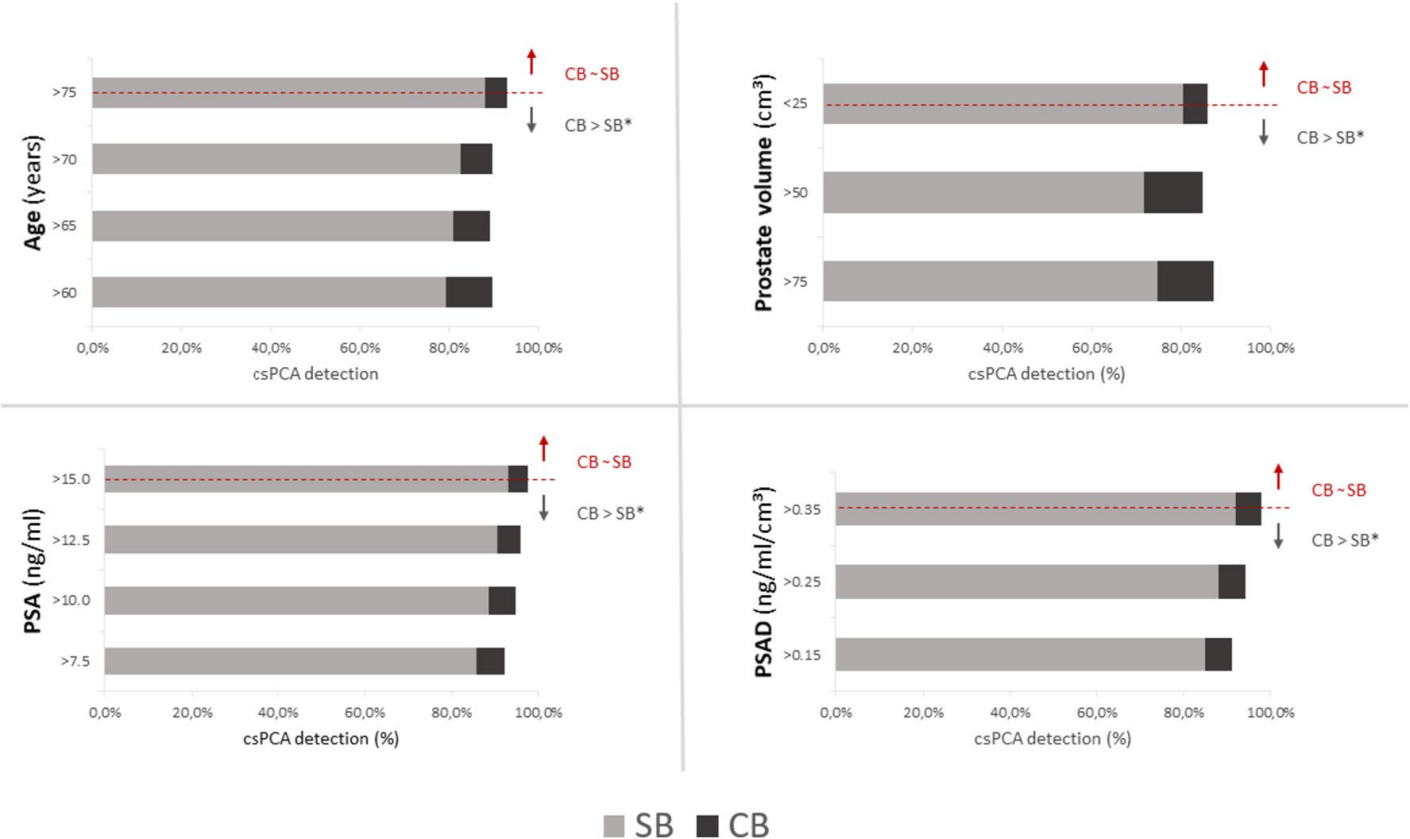
csPCA detection by CB and SB dependent on PSA, PSAD, prostate volume and age. Figure illustrates the identification of threshold values, represented by the dotted red line, for age, prostate volume, prostate-specific antigen (PSA), and prostate-specific antigen density (PSAD), which are indicative of comparable diagnostic efficacy for the combined biopsy approach (CB) and the systematic biopsy approach (SB), as denoted by the red arrow. However, when these clinical parameters fall below the established cut-off values, the performance of CB exceeds that of SB in detecting cases of clinically significant prostate cancer, as shown by the gray arrow, with statistical significance at *p* < 0.05 = *. This is demonstrated in the graph by the amplification of the black bar segments and/or reduction in the gray elements

*By* comparing SB and CB performance, we found that CB is superior concerning csPCA detection in the vast majority of cases (72.7% vs 60.6%, respectively). This superiority was also confirmed at high PSA (> 15 ng/ml), high PSAD levels (> 0.5 ng/ml/cm^3^) and abnormal DRE findings (all *p* < 0.001). But in patients with higher tumor burden, indicated by clinical and laboratory surrogate markers, the incremental value of the additive TB was less pronounced. In men with both suspicious DRE and either high levels of PSA (> 12.5 ng/ml), PSAD > 0.35 ng/ml/cm^3^, smaller prostate volume (< 25cm^3^), or increased age (> 75 years) statistical equivalence between SB and the CB was observed (*p* = 0.063, *p* = 0.063, *p* = 0.125, *p* = 0.096, respectively). In particular, among men aged > 75 years who had an abnormal DRE, the value of performing an additional TB over performing SB only is not superior, even when using lower cut-offs (PSA ≥ 10.0 ng/ml and PSAD ≥ 0.15 ng/ml/cm^3^). If these thresholds are applied, it comes at the expense of missing ~ 5% of csPCA cases in these particular subgroups, Fig. 1.

## Discussion

To date, no single biopsy method comprehensively covers the diagnostic spectrum of PCA. Hence, a strategic allocation of resources through subgroup delineation is essential. Particularly, when diagnosing clinically evident PCA, the prevailing dogma of mandating an MRI prior to every biopsy cannot endure. Our study underscores the efficacy of cost-effective clinical parameters, including PSAD and DRE, in judiciously applying targeted biopsy techniques. This aligns with previous findings showing PCA sensitivity of up to 90% in cases of concurrently elevated PSA, suspicious DRE, and TRUS findings [18].

On the other hand, consistent with previous findings, our study revealed a significant increase in the detection rates of both PCA and csPCA with the utilization of CB as compared to either SB or TB across the vast majority of men [19–23]. Hereby, the current csPCA detection was markedly increased (72.7%) compared to other series [1–3, 22]. This can be attributed to selection bias, as the study only included men who had confirmed PCA and MRI-positive results. This might also explain the comparable diagnostic performance of TB and SB for csPCA in our cohort, which is not consistent with previous findings. Nonetheless, the results corroborate prior prospective series that have demonstrated the superior efficiency of TB in terms of CDR per core taken and csPCA-detection in PI-RADS 5 rated patients, despite detecting significantly fewer nsPCA cases [2, 3, 22]. Notably, in biopsy-naïve PI-RADS-3-rated men, the necessity of performing additive SB was emphasized, as it led to a significant increase in csPCA detection by nearly 10% (47.0% vs. 37.6%). In addition, our findings support the previously established benefit of utilizing the transperineal route as opposed to the transrectal biopsy route. However, the supplementary diagnostic value conferred by the utilization of both targeted and systematic biopsy approaches remains pertinent irrespective of the chosen biopsy route [24].

Moreover, our findings suggest a considerable level of underestimation in the accurate grading of tumors when using single biopsy methods, with a mismatch observed in over 50% of cases. This supports previous research that has highlighted the superior concordance of CB with pathological tumor grading, emphasizing the significance of performing both SB and TB to reduce the risk of misdiagnosis [19, 25].

Despite experienced investigators performing TB in high-volume tertiary centers in accordance with current recommendations, which includes taking at least 3 cores on average, our study shows a slightly higher degree of underestimation in the diagnosis of csPCA with a TB-only approach, missing 9.4% csPCA, compared to what was reported in prior prospective studies (missing 4.9–5.8% of cases [4, 19, 22]). However, these results align with Drost et al.’s systematic review, where the TB alone strategy in MRI-positive men missed the diagnosis in 17.2% of men with ISUP grade 2 or higher PCA [4]. Furthermore, utilizing only SB would have led to an underestimation of csPCA grade in 14.2%, 18.6% and 14.5% of men with PI-RADS ratings of 3–5. Taken together, these findings highlight the superiority of the recommended combined approach. Hence, the costs and time required to provide pre-interventional MRI are justified for the majority of patients with suspected PCA, as the consequences of unreliable risk stratification at the outset of treatment may result in unnecessary morbidities that far outweigh the costs of achieving an accurate diagnosis [26]. It is therefore advisable to strive toward removing obstacles for patients and healthcare systems, to enhance accessibility to advanced techniques like MRI. In this context, the discussion should encompass innovative imaging alternatives such as multiparametric ultrasound (mpUS), micro-ultrasound (MUS), and artificial intelligence ultrasound (AIUS). These technologies enable a direct, targeted biopsy, eliminating the need for indirect fusion and reducing the risk of communication errors and image processing issues in interdisciplinary collaboration. In the case of MUS-TB, a comparable detection rate to MRI-TB for csPCA was demonstrated, concurrently reducing overdiagnosis of nsPCA [27, 28]. However, substantial acquisition costs do not present a clear financial advantage over MRI. A more cost-effective approach is mpUS. However, based on the prospective CADMUS study, mpUS identified fewer csPCa than MRI. Hence, mpUS-TB might find application in patients suspected of having PCA when MRI is not available [29]. A limitation of both approaches is their high variability among operators [30]. Another solution involves decentralized AIUS-TB, requiring no additional equipment and ensuring operator independence [31]. Recently, a study demonstrated promising csPCA detection rates, sparing unnecessary cores in a randomized controlled setting [32].

Notably, our analysis revealed that irrespective of the biopsy method, csPCA detection is associated with clinical and biochemical surrogate markers like PSA, PSAD and DRE [33, 34]. However, in multivariate analysis, only abnormal DRE was a significant predictor of csPCA. Despite the recent disqualification of DRE as a useful screening tool, this finding suggests that performing DRE in men suspected of having PCA can provide a high incremental value in effectively distinguishing those with csPCA [35].

Additionally, we were able to pinpoint a specific cohort of patients with increased risk of csPCA based on elevated PSA levels (PSA > 12.5 ng/ml, or PSAD > 0.35 ng/ml/cm^3^) and positive DRE results, who may forego pre-biopsy MRI with acceptable levels of diagnostic uncertainty. Two potential strategies could be considered here: Firstly, elderly patients (> 75 years) may be able to skip pre-interventional MRI at lower PSA and PSAD thresholds. Secondly, for men with PSA levels > 20 ng/ml and abnormal palpation, performing SB only could be a justifiable approach, reducing the need for expansive diagnostic procedures before a prostate biopsy. Given these findings, foregoing pre-interventional MRI to prevent diagnosis delay, which potentially causes psychological distress, may be a reasonable approach, especially in resource-limited regions with insufficient infrastructure [36]. However, such a decision should be based on careful consideration of available resources and potential drawbacks.

In spite of its limitations such as its retrospective nature, our study lacked information on the final tumor grade obtained from prostatectomy specimens. Furthermore, the thresholds established in our study were derived from a MRI-selected cohort with confirmed tumor presence, which cannot be extrapolated directly to the general population. However, this study, involving over 2700 participants from multiple centers, confirms previous observations that recommend a combined biopsy approach in most cases, irrespective of the biopsy route, as it provides an optimized risk stratification. Nevertheless, a specific subgroup of men with an increased risk of csPCA based on abnormal DRE and elevated PSA levels may not benefit significantly from incorporating a costly pre-interventional MRI. Therefore, in resource-limited settings, it may be acceptable to take calculated risks and opt for an early SB instead of using MRI in this particular subset. Establishing a phased diagnostic process with diverse biopsy methods is essential for efficient resource allocation, identifying straightforward cases for decentralized completion, and reserving capacity in specialized centers for complex cases.

## Conclusions

Performing a combined biopsy offers the highest diagnostic accuracy and reduces the risk of underdiagnosis in most men without a previous biopsy. However, in patients with abnormal DRE and elevated PSA levels (PSA > 12.5 ng/ml, or PSAD > 0.35 ng/ml/cm^3^), and/or advanced age, using only a systematic biopsy can yield similar rates of detecting csPCA. Therefore, if resources are limited, a modified biopsy approach may be considered for these individuals.

## Data Availability

The data that support the findings of this study are available from the corresponding author upon reasonable request. Comprehensive data supporting the main findings of the study are additionally presented in the supplementary material.
